# Altered sialin mRNA gene expression in type 2 diabetic male Wistar rats: implications for nitric oxide deficiency

**DOI:** 10.1038/s41598-023-31240-4

**Published:** 2023-03-10

**Authors:** Nasibeh Yousefzadeh, Sajad Jeddi, Maryam Zarkesh, Khosrow Kashfi, Asghar Ghasemi

**Affiliations:** 1 Endocrine Physiology Research Center, Research Institute for Endocrine Sciences, Shahid Beheshti University of Medical Sciences, No. 24, Arabi Street, Daneshjoo Blvd, Velenjak, P.O. Box 19395-4763, Tehran, Iran; 2 Cellular and Molecular Endocrine Research Center, Research Institute for Endocrine Sciences, Shahid Beheshti University of Medical Sciences, Tehran, Iran; 3 Department of Molecular, Cellular, and Biomedical Sciences, Sophie Davis School of Biomedical Education, City University of New York School of Medicine, New York, NY USA

**Keywords:** Metabolism, Transcription

## Abstract

Nitrate therapy has been suggested to boost nitric oxide (NO) levels in type 2 diabetes (T2D); however, little is known about nitrate transport across the membranes. This study aimed to assess changes in the mRNA expression of sialin, as a nitrate transporter, in the main tissues of rats with T2D. Rats were divided into two groups (n = 6/group): Control and T2D. A high-fat diet combined with a low dose of streptozotocin (STZ, 30 mg/kg) was used to induce T2D. At month 6, samples from the main tissues of rats were used to measure the mRNA expression of sialin and levels of NO metabolites. Rats with T2D had lower nitrate levels in the soleus muscle (66%), lung (48%), kidney (43%), aorta (30%), adrenal gland (58%), epididymal adipose tissue (eAT) (61%), and heart (37%) and had lower nitrite levels in the pancreas (47%), kidney (42%), aorta (33%), liver (28%), eAT (34%), and heart (32%). The order of sialin gene expression in control rats was: soleus muscle > kidney > pancreas > lung > liver > adrenal gland > brain > eAT > intestine > stomach > aorta > heart. Compared to controls, rats with T2D had higher sialin mRNA expressions in the stomach (2.1), eAT (2.0), adrenal gland (1.7), liver (8.9), and soleus muscle (3.4), and lower sialin expression in the intestine (0.56), pancreas (0.42), and kidney (0.44), all *P* values < 0.05. These findings indicate altered sialin mRNA expression in the main tissues of male T2D rats and may have implications for future NO-based treatment of T2D.

## Introduction

The prevalence of type 2 diabetes (T2D) in adults increased from 151 to 537 million during the last two decades, and it is estimated to reach 783 million by 2045^1^. Decreased nitric oxide (NO) bioavailability, the amount of NO that becomes available to its targets, is involved in the pathophysiology of T2D^2^. NO is produced via NO synthase (NOS)-dependent and NOS-independent (nitrate-nitrite-NO) pathways^3^ that account for about 90% and 10% of the whole-body NO production, respectively^4^. In the NOS-dependent pathway, NO is synthesized from L-arginine. In the second pathway, NO is produced from the reduction of nitrate and nitrite^5^, where nitrate has endogenous (oxidation of NOS-derived NO) and exogenous (mainly through diet) sources^4,6^. The nitrate-nitrite-NO pathway is complementary to the NOS-dependent pathway^7,8^. In support, dietary nitrate/nitrite deprivation leads to decreased skeletal muscle nitrate and nitrite in rats^7^ and developing metabolic syndrome and endothelial dysfunction in mice^9^. In T2D, both NO production pathways are disrupted, and decreased endothelial NOS (eNOS) and increased inducible NOS (iNOS) expression and activity^10,11^ and impaired nitrate-nitrite-NO pathway^12^, have been reported.

Intervention by nitrate and nitrite, which have NO-like bioactivity^5^, is now one strategy for NO boosting in NOS-disrupted conditions, including T2D^5,13^. Indeed, the beneficial metabolic effects of nitrate/nitrite in rodent models of T2D have been shown^14,15^, and the underlying mechanisms include increased insulin secretion from pancreatic β-cells^16,17^ as well as improved peripheral glucose utilization^15,18–21^. Stimulatory effect of nitrate/nitrite on insulin secretion in rats with T2D are mediated by increasing pancreatic islets' blood flow^16^, increasing pancreatic islets' insulin synthesis and exocytosis^17^, and blunting diabetes-induced oxidative stress in pancreatic islets^22^. Despite extensive research on the beneficial effects of nitrate and nitrite in T2D, little is known about nitrate transport across the membranes. Nitrate cannot freely permeate through the phospholipid bilayer and needs transporters to move across the plasma membrane^23^.

Slc17a5 (solute carrier family 17, member 5) gene that encodes sialin protein, was first identified in 1999^24^. Slc17a5 is located on chromosome 6q in humans and 9q in rodents^25^ and is a highly conserved gene in humans and rodents^26^. The Slc17 family includes type I phosphate, sialin, vesicular glutamate, and vesicular nucleotide transporters^27,28^. Sialin protein has 495 amino acids^29^ and, in both humans and rodents, is an integral membrane protein that has 12 transmembranes domains^25,30^. In 2012, it was reported that sialin could act as a 2NO_3_^−^/H^+^ cotransporter in the salivary glands, which causes nitrate influx to the cell^31^. Nitrate transport into cells through sialin is essential for the nitrate-nitrite-NO pathway^32^. Sialin is widely expressed in the eye, brain, liver, pancreas, kidney, muscles, and salivary glands^31,33–35^. This wide expression in almost all tissues in both humans and rodents^28^ reflects a housekeeping function for this carrier^27^. Inhibition of sialin or its knockout decreases nitrate uptake in human skeletal muscle cells^36^ and human submandibular gland cell line (HSG), whereas its overexpression increases nitrate uptake^31^. In addition, sialin expression increases in hypoxic cancer cells^37^ and hypercholesterolemic rats' heart and liver tissues^38^. To our knowledge, changes in sialin gene expression, if any, have not been reported in T2D. Therefore, this study aimed to assess the changes in sialin mRNA expression in the main tissues of type 2 diabetic rats.

## Materials and methods

### Ethical approval

All experiments of the current study were affirmed by the published guideline of the care and use of laboratory animals in Iran^39^ and reported following ARRIVE guidelines^40^. The ethics committee of the Research Institute for Endocrine Sciences affiliated with the Shahid Beheshti University of Medical Sciences confirmed and approved all experimental procedures of the current study (Ethic Code: IR-SBMU.ENDOCRINE.REC.1398.034; Approved Date: 2019-08-06).

### Induction of type 2 diabetes in rat

Male Wistar rats (n = 12), 2 months old, weighing 190–200 g, were housed in polypropylene cages under standard conditions with free access to regular rat diet (Khorak Dam Pars, Co., Tehran, Iran) and drinking water. Rats were randomly allocated to 2 groups (n = 6/group): Control and T2D. A high-fat diet (HFD) combined with streptozotocin at a low dose (STZ, 30 mg/kg, intraperitoneal (IP) injection) was used to induce T2D; one week later, anesthetized rats with overnight (12 h) fasting serum glucose concentration ≥ 150 mg/dL were included in the study as diabetic rats^41^. For the preparation of HFD, 586 g of powdered regular diet, 310 g of sheep butter as a source of fat, 73 g of casein (Iran Caseinate Company, Karaj, Iran) as a source of protein, 1.8 g, 4.1 g, and 25 g of DL-methionine, vitamin mix, and mineral mix (Behroshd Company, Saveh, Iran) were thoroughly mixed to produce 1000 g HFD. In the prepared HFD, the total caloric value was ~ 4900 kcal/1000 g, and calories received from fat, carbohydrate, and protein were 58.8%, 27.0%, and 14.2%, respectively. Details on the induction of T2D in rats using combination of HFD and low dose of STZ have been reviewed in our previous report^41^.

### Experimental design

**Figure 1:**
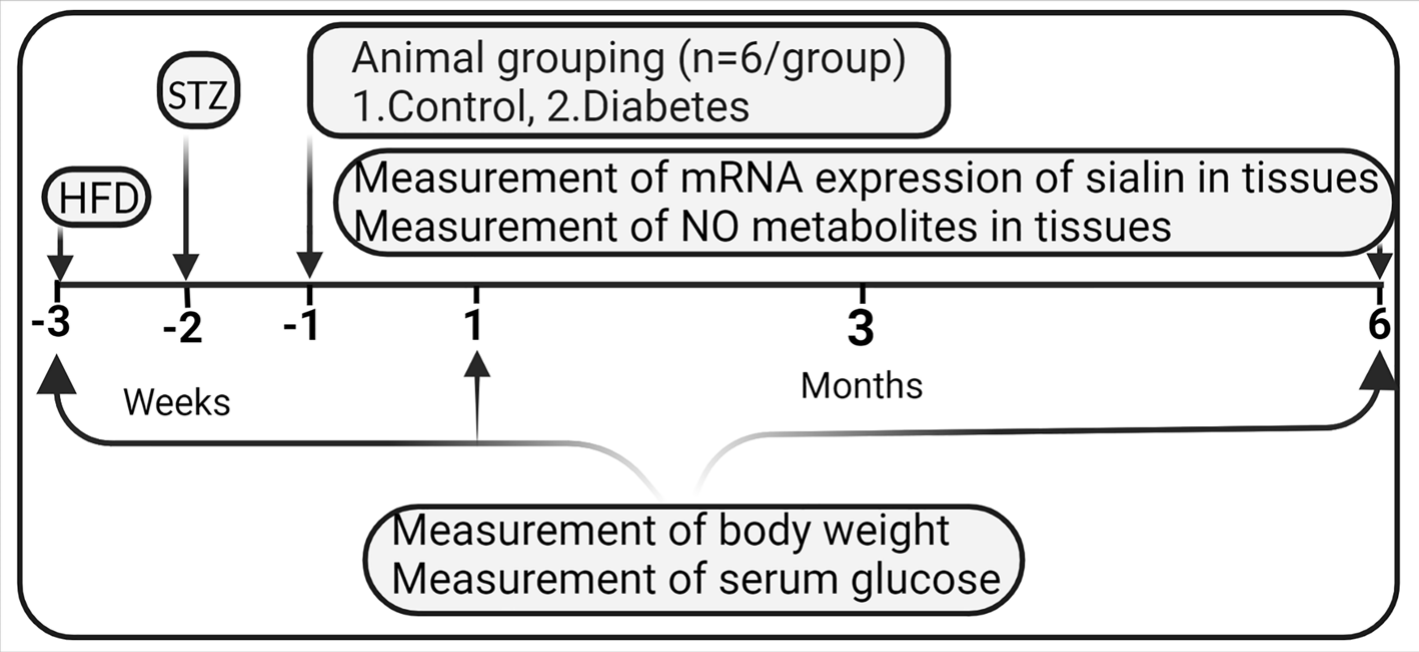
Experimental design of the study. *HFD* high fat diet, *STZ* streptozotocin, *NO* nitric oxide.

The protocol for this experimental interventional study is shown in Fig. 1. At month 0 (start of the experiment) and month 6 (end of the experiment), body weight (using Tefal Scale; sensitivity 1 g) and serum glucose were measured in all rats. At month 6, samples from main tissues, including the left ventricle of the heart, aorta, stomach, intestine (i.e., duodenum), epididymal adipose tissue (eAT), brain, adrenal gland, liver, lung, pancreas, kidney, and soleus skeletal muscle were used to measure the mRNA expression of sialin (Slc17a5) using real-time PCR. In addition, NO metabolites (nitrate + nitrite = NOx) in all studied tissues were measured at month 6 by the Griess method, as previously reported^42^.

### Measurement of serum glucose concentration

Serum glucose concentration was measured at months 0 and 6 by the glucose oxidase method (Pars Azmoon Co., Iran). After overnight fasting (12 h), blood samples were obtained from the tail tips of anesthetized rats (IP injection of ketamine at dose 50 and xylazine at dose 10 mg/kg) and centrifuged (10 min at 5000*g*) and then the sera were used to measure serum glucose concentrations; the intra-assay coefficient of variation (CV) was 2.6%.

### Measurement of tissues’ nitric oxide metabolites

The NOx and nitrite concentration in all tissues were measured by the Griess method^42^, with slight modification on sample deproteinization. According to our previous report^43^, to deproteinize samples, we used zinc sulfate (15 mg/mL) instead of centrifugation by a 30-kDa molecular weight filter as reported by Miranda et al.^42^; in addition, NaOH (3.72 M^44^) was used for preventing turbidity in the Griess reaction. After adding zinc sulfate and NaOH to 300 μL of the homogenized tissues, samples were centrifuged (10 min at 10,000*g*), and supernatants were removed o measure of NOx and nitrite levels. In brief, all tissues were homogenized (100 mg of the heart, aorta, stomach, intestine, adrenal gland, liver, lung, pancreas, kidney, and soleus skeletal muscle in 500 μL of phosphate-buffered saline (PBS, pH 7.4) and 100 mg of the brain and eAT in 200 μL of PBS) and centrifuged for 10 min at 10,000 g. For measuring NOx, nitrate was reduced to nitrite by adding vanadium trichloride (VCL3, 8 mg/mL in 1 M HCl, the solutions passed through the membrane filter), followed by N-(1-naphthyl) ethylenediamine (0.1% in ddH_2_O) and sulfanilamide (2% in 5% HCl). Samples were kept for 30 min at 37 °C, and optical density (OD) was read at 540 nm. NOx concentrations were measured using a standard calibration curve of 0–100 µM sodium nitrate.

Nitrite was measured using the same method, except that samples were only exposed to sulfanilamide, NEDD, and 1 M HCl instead of VCL3; nitrite standards (0–20 µM) were used. Tissue nitrate concentrations were calculated by subtracting nitrite values from NOx concentrations. Protein concentration was measured using the Bradford method; nitrite and nitrate concentration in tissues are presented as nmol/mg protein. The intra-assay CVs for NOx in the heart, aorta, stomach, intestine, adrenal gland, liver, lung, pancreas, kidney, brain, eAT, and soleus skeletal muscle were 2.2, 1.7, 2.7, 2.0, 1.4, 2.2, 2.1, 2.5, 2.9, 2.9, 1.8, and 3.5%, respectively. The intra-assay CVs for nitrite in the heart, aorta, stomach, intestine, adrenal gland, liver, lung, pancreas, kidney, brain, eAT, and soleus skeletal muscle were 3.0, 3.6, 2.0, 2.8, 2.8, 4.0, 2.2, 1.5, 2.9, 2.3, 2.5, and 3.2%, respectively.

### Assessment of sialin mRNA expression

Total RNA from all tissues were extracted using the TRIzol reagent (Invitrogen, USA). The purity and quantity of the extracted total RNA was determined using A NanoDrop-1000 spectrophotometer (Thermo Scientific, USA). The 260/280 and 260/230 absorbance ratios were used as indices of RNA purity; a pure RNA sample (i.e., free of protein and phenolic compounds and other solvent contamination) is characterized by 260/280 and 260/230 absorbance ratios ranging between 1.8 and 2.2^45^. For cDNA synthesis, a cDNA synthesis kit (SMOBiO Technology, Taiwan), in accordance with manufacturer instructions, and a peqSTAR Universal PCR machine (Peqlab, Germany) was used. The reaction contained 1 μg of extracted RNA, 1 μL of ExcelRT Reverse transcriptase (RT) (200 U/μL), 4 μL of RT buffer, 1 μL of random hexamer (100 µM), 1 μL of dNTPS Mix (10 mM), 1 μL of RNAok RNase inhibitor (20 U/μL) and 4 μL of DEPC-treated H_2_O. The thermal cycling settings included 5 min at 70 °C for RNA denaturation followed by incubation at 25 °C for 20 min and 50 °C for 50 min, respectively.

Finally, amplification of synthesized cDNA was done using SYBR Green PCR Master Mix 2X (Ampliqon Company, Denmark) in a Rotor-Gene 6000 real-time PCR machine (Corbett, Life Science, Sydney, Australia). The reaction contained 2 µL cDNA, 2 μL of primers (forward and reverse), 12.5 μL Master Mix, and 8.5 μL DEPC-treated H_2_O, yielding a total volume of 25 µL. The thermal cycling settings included a 10 min initial denaturation (95 °C) followed by 40 cycles with 45, 45, and 60 s at 94, 58, and 72 °C, respectively. All tissues were run in duplicate; nuclease-free water was used instead of templates in the negative control reactions. Sequences of primer for sialin and GAPDH (housekeeping) genes are presented in Table 1. We used GAPDH as a housekeeping gene because it is useful when the study aims to compare gene expression between tissues because its expression is abundant and less variable among tissues^46^. GAPDH has relatively stable expression in the liver^47^, kidney^48^, pancreas^49^, heart^50^, brain^51^, adipose tissue^47^, and intestine^52^ in rodents. In addition, its expression remains relatively stable in the tissues of rodents with T2D^53^.

**Table 1:** Primers sequence used for Real time-PCR.

Genes	Accession no	Sequence (5' → 3')	PCR product length (bp)
Sialin	NM_001009713.2	F: GTCAGCCAAGCAACGATAG R: AAGCATAGAGAACGAAGAAACC	209
GAPDH	NM_017008.4	F: AGTGCCAGCCTCGTCTCATA R: GATGGTGATGGGTTTCCCGT	248

*F* forward, *R* reverse.

### Statistical analyses

Data were analyzed using GraphPad Prism version 8.0.0 for Windows, GraphPad Software, San Diego, California USA, www.graphpad.com. All values are presented as mean ± SEM, except for mRNA expressions of sialin, which are represented as relative fold changes. A two-way mixed (between-within) analysis of variance followed by the Bonferroni post-hoc test was used to compare body weight and glucose at the start and end of the study among control and T2D rats. The Student's t-test was used to compare the NOx, nitrite, and nitrate levels between groups. To determine the precision of the assays, CV was calculated using the formula: CV = (standard deviation/mean) × 100)]^54^. Relative expressions of genes were calculated based on cycle thresholds of sialin versus GAPDH as a reference gene using the REST software^55^. This software uses a randomization test to compare the difference between control and diabetic samples, which avoids any assumptions about data distribution and is therefore preferred over parametric tests^55^. Quantities of interest in PCR data are derived from ratios and variances can be high, so standard parametric tests, which depend on normal distribution, are inappropriate for their statistical analysis. A randomization test repeatedly and randomly reallocates the observed values to control and treated groups and notes the expression ratio. *P* values are calculated from the proportion of random allocations of mean observed data to the control and treated groups. Since examining of all possible allocations is impractical, a random sample is drawn; taking 2000 samples or more provides a reliable estimate of *P* value at 0.05 level^55^. In addition, REST software provides efficiency-corrected relative gene expression, which is highly recommended^55^, prevents any miscalculating expression ratio differences^56^, and allows the comparison of two groups for one reference and one or more target genes^55^. Two-sided *P *values < 0.05 and between 0.05 and 0.10 were considered statistically and marginally significant, respectively.

## Results

### Serum glucose and body weight

As shown in Table 2, compared to month 0, both control and diabetic rats had higher body weight by 84% and 113% (*P* < 0.001) at month 6. In addition, compared to month 0, serum glucose levels at month 6 were higher by 57% (*P* < 0.001) in the diabetic rats.

**Table 2:** Body weight and fasting serum glucose concentration at the start and end of the study in control and type 2 diabetic rats.

	Control		Diabetes	
	Month 0	Month 6	Month 0	Month 6
Body weight (g)	192.8 ± 3.6	354.4 ± 7.3*	186.2 ± 6.2	396.3 ± 7.8*
Fasting glucose (mg/dL)	117.2 ± 6.9	127.5 ± 3.8	110.2 ± 4.2	172.7 ± 7.4*

*Significant difference compared to month 0. Values are mean ± SEM. (n = 6 rats/group).

### Tissue nitrate and nitrite levels

**Figure 2:**
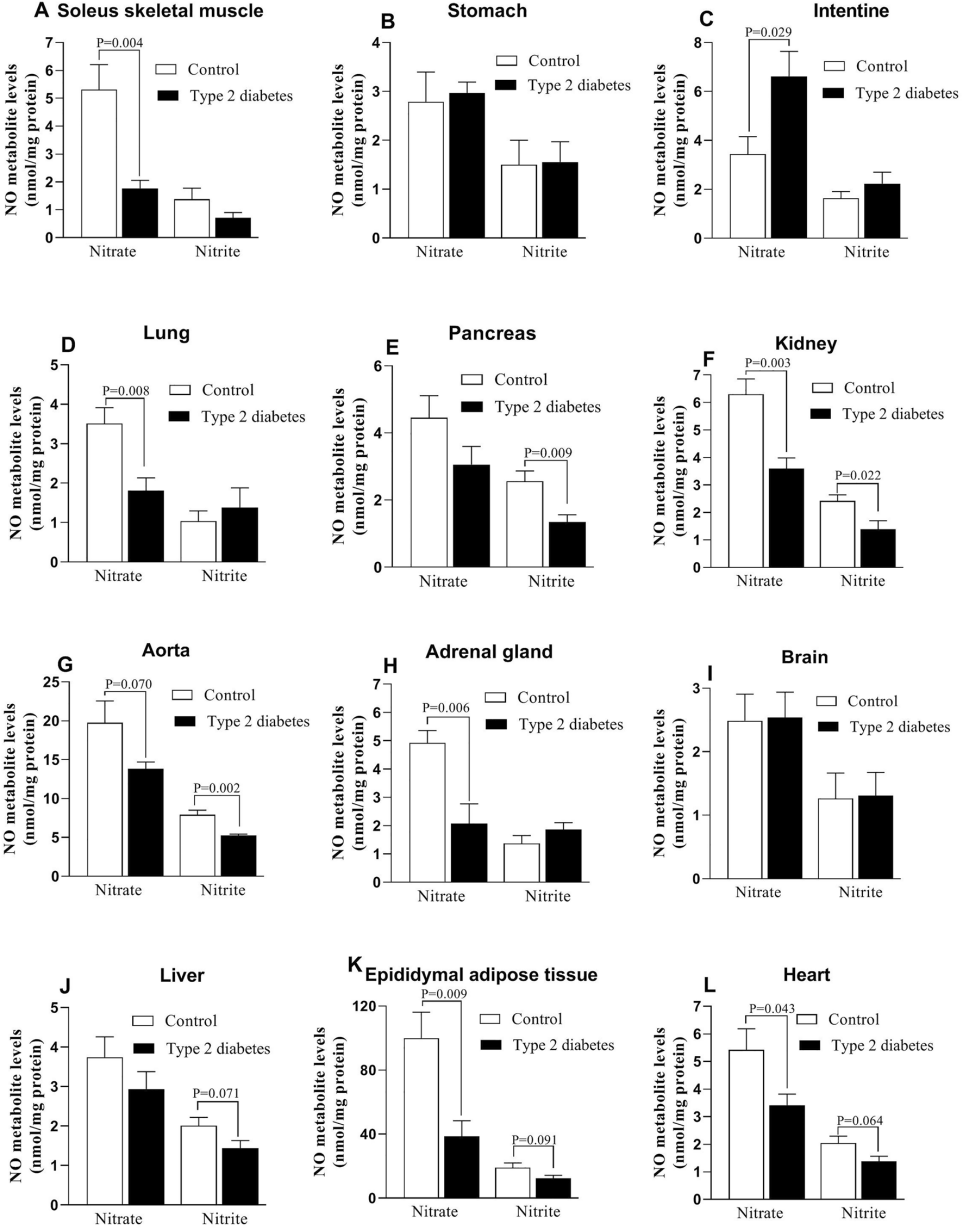
Changes in tissues’ nitrate and nitrite levels in control and type 2 diabetic rats.

As presented in Fig. 2, compared to controls, rats with T2D had lower values of nitrate in the soleus muscle (66%, *P* = 0.004), lung (48%, *P* = 0.008), kidney (43%, *P* = 0.003), aorta (30%, *P* = 0.070), adrenal gland (58%, *P* = 0.006), eAT (61%, *P* = 0.009), and heart (37%, *P* = 0.043); however, nitrate levels were higher in the intestine of diabetic rats (92%, *P* = 0.029) and no changes were observed in the stomach, pancreas, brain, and liver.

Compared to controls, rats with T2D had lower values of nitrite levels in the pancreas (47%, *P* = 0.009), kidney (42%, *P* = 0.022), aorta (33%, *P* = 0.002), liver (28%, *P* = 0.071), eAT (34%, *P* = 0.091), and the heart (32%, *P* = 0.064). Nitrite values in the soleus muscle, stomach, intestine, lung, adrenal gland, and brain were similar among control and diabetic rats.

### Sialin mRNA expression in tissues

**Figure 3:**
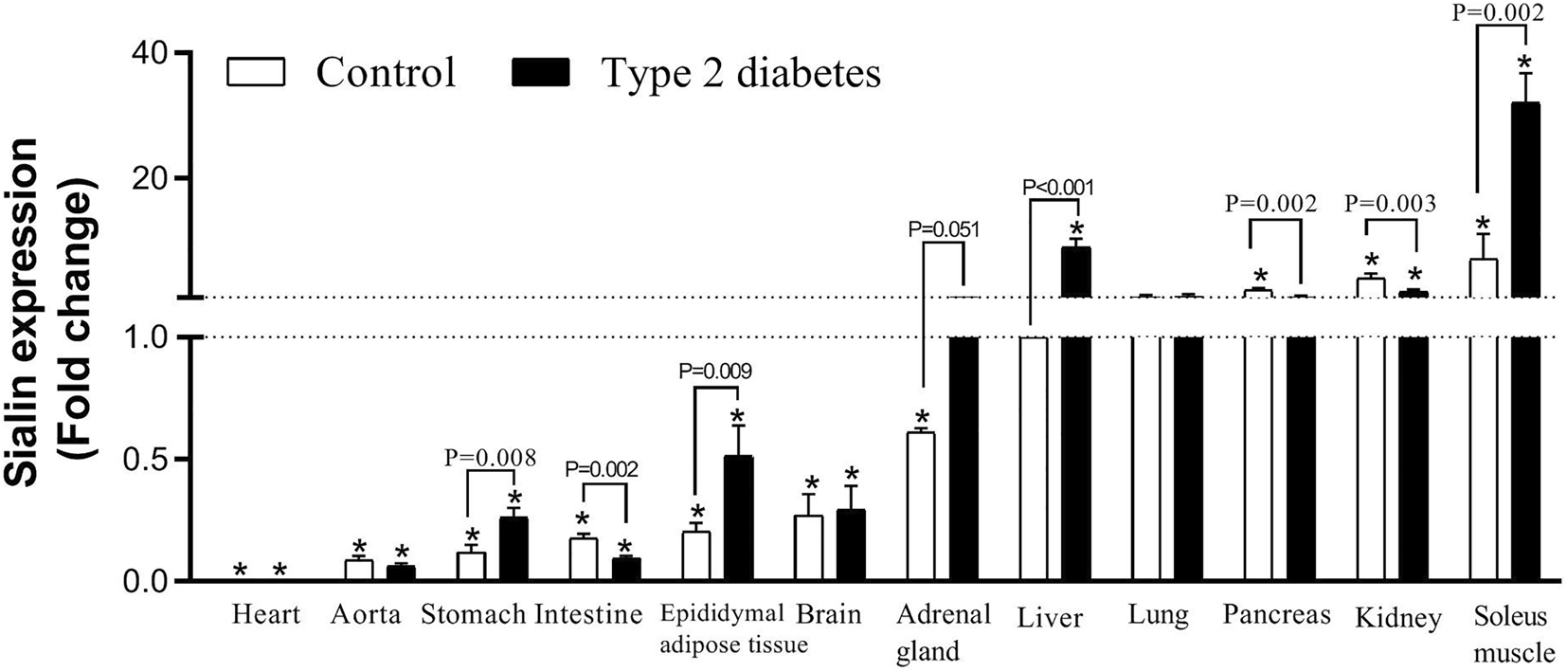
Changes in mRNA expression of sialin in control and type 2 diabetic rats. *Significant difference compared to the liver tissue of control rats, which its expression was considered as reference.

As shown in Fig. 3, compared to the control group, rats with T2D had higher mRNA expressions of sialin in the stomach, eAT, adrenal gland, liver, and soleus muscle by 2.1, 2.0, 1.7, 8.9, and 3.4 folds, respectively. mRNA expressions of sialin were significantly lower in the intestine, pancreas, and kidney of the diabetic rats compared to controls by 0.56, 0.42, and 0.44 folds, respectively. No change was observed in the mRNA expression of sialin in the heart, aorta, brain, and lung of diabetic rats (Supplementary Figure 1).

If mRNA expressions of sialin in the liver of control rats is considered as reference (sialin mRNA expression = 1), in control rats, mRNA expressions of sialin in the heart (0.006-fold), aorta (0.089-fold), stomach (0.11-fold), intestine (0.17-fold), eAT (0.2-fold), brain (0.27-fold), and adrenal gland (0.61-fold) were lower than the liver; all *P* values are < 0.001. Compared to the liver, sialin mRNA expression was higher in the pancreas (2.17-fold, *P* < 0.001), kidney (4.15-fold, *P* < 0.001), and soleus muscle (7.17-fold, *P* < 0.001). mRNA expressions of sialin in the lung of rats were comparable with the liver.

Compared to mRNA expressions of sialin in the liver of controls, rats with T2D had higher sialin mRNA expression in the soleus muscle (32.1-fold, *P* < 0.001) and kidney (1.97-fold, *P* = 0.005) and lower expression in the heart (0.008-fold, *P* < 0.001), aorta (0.063-fold, *P* < 0.001), stomach (0.26-fold, *P* < 0.001), intestine (0.096-fold, *P* < 0.001), eAT (0.51-fold, *P* = 0.010), and brain (0.29-fold, *P* < 0.001). No change was observed in the sialin mRNA expressions in the adrenal gland, lung, and pancreas of diabetic rats, as compared with the expressions in the liver of controls.

## Discussion

To our knowledge, this is the first report documenting changes in mRNA expression of sialin, as a nitrate transporter, in the main tissues of rats with T2D. mRNA expression of sialin in the control rats differed among tissues, with the lowest expression registered in the heart and the highest in the soleus muscle. T2D increased mRNA expression of sialin in most tissues (soleus muscle, stomach, adrenal gland, liver, and eAT), decreased it in some tissues (intestine, pancreas, and kidney), and did not affect other studied tissues (lung, aorta, brain, and heart).

Our results indicate that except for the brain, stomach, and intestine, in other studied tissues, lower values of nitrate (soleus muscle, lung, kidney, aorta, adrenal gland, eAT, and heart) or nitrite (pancreas, kidney, aorta, liver, eAT, and heart) are observed in diabetic rats. These results represent a generalized decreased NO bioavailability in T2D and are in line with other reports indicating lower NOx concentrations in the liver (43%)^57^, soleus muscle (64%)^57^, eAT (42%)^57^, inguinal adipose tissue (30%)^15^, kidney (42%)^58^, heart (60%)^58^, and aorta (40%)^59^ of rats with T2D as well as lower nitrate (33%) and nitrite (51%) concentrations in the gastrocnemius muscle^18^ and heart (43%)^60^ of mice with T2D. In addition, chronic metabolic conditions, including hypertension and T2D, are characterized by reduced NO production^61^. Decreased NO metabolites in tissues of diabetic rats are due to impaired NOS-dependent NO production, including decreased availability of L-arginine, decreased eNOS expression, increased arginase activity, uncoupling of NOS, and increased levels of asymmetric dimethyl L-arginine (ADMA, an endogenous NOS inhibitor)^57,62^. In T2D, eNOS is uncoupled and produces superoxide anion instead of NO^62^. Superoxide anion rapidly reacts with iNOS-derived NO to form peroxynitrite, a potent oxidant, which enhances eNOS uncoupling^62,63^ by increasing intracellular ADMA levels (an L-arginine analog) and oxidation of tetrahydrobiopterin (an eNOS cofactor)^62,64,65^. In addition, oxidation of eNOS by peroxynitrite^66^, may be an essential mechanism in the development of eNOS uncoupling and decreased NO metabolites in diabetic conditions. The impaired nitrate-nitrite-NO pathway is also involved in NO deficiency in T2D and is due to decreased reduction of nitrate to nitrite and then to NO because of oral microbiota dysbiosis, which decreases oral nitrate-reducing bacteria, abnormal metabolism of ascorbic acid, which decreases gastric conversation of nitrite to NO, and decreased nitrate-nitrite reductase enzymes^67–69^.

In our study, the order of sialin expression in tissues of control rats (soleus muscle > kidney > pancreas > lung > liver > adrenal gland > brain > eAT > intestine > stomach > aorta > heart) was different from those reported for humans (brain > kidney > liver > pancreas) and pigs (liver > brain > kidney > muscle > pancreas)^31^, indicating species difference in the tissue pattern of sialin expression. These findings are supported by data showing species differences in NO metabolism; it has been reported that NO production in the rat (0.55 ± 0.05 μmol/kg/h) is similar to humans (0.38 ± 0.06 μmol/kg/h) but it is about 20 times higher in mice (7.68 ± 1.47 μmol/kg/h)^4,61^. In addition, the accumulation of nitrate in the saliva (with a concentration 10–20 fold higher than blood) in humans is more effective than in rodents; however, nitrate reduction in basal conditions in rodents is more effective than humans^4,61^.

Compared to the controls, in rats with T2D, sialin mRNA expression was higher in the adrenal gland, eAT, stomach, soleus muscle, and liver. Conversely, it was lower in the intestine, pancreas, and kidney. In most studied tissues, increased sialin mRNA expression in diabetic rats was accompanied by decreased nitrate and nitrite concentrations, suggesting that increased sialin mRNA expression acts as a compensatory mechanism to counteract reduced NO bioavailability. In support of this notion, it has been reported that sialin expression in the skeletal muscle of control mice increased by 89% upon deletion of myoglobin, which reduces nitrite to NO^70^, and this compensatory pathway partially sustains NO bioavailability in myoglobin-deficient mice^70^. Moreover, it has been reported that a decrease in dietary nitrate for 21 days that causes decreased nitrate levels in the soleus muscle is associated with increased sialin expression by 50% in rats^71^.

In the current study, sialin mRNA expression was lowest in the heart and highest in the soleus muscle. It has been reported that NO production in the heart of normal Wistar rats mainly relies on the NOS-dependent rather than the nitrate-nitrite-NO pathway; indeed, NOS-dependent and -independent pathways contribute about 80% and 20% to the total heart NO formation, respectively. However, in the ischemic heart, NO formation from the NOS-independent pathway increases and can exceed NOS-dependent NO generation^72^. Our finding that sialin mRNA expression was highest in the skeletal muscle is in line with the hypothesis proposed by Piknova et al. that skeletal muscle tissue is the main nitrate reservoir organ and the liver and other organs are the final site of reduction of nitrate to nitrite and then NO^73^. According to this hypothesis, due to its large size and low nitrate reductase activity, skeletal muscle is an optimal place to store nitrate and may have a protective role against possible future periods of dietary nitrate deprivation, at least in rodents^73^.

In our study, decreased sialin mRNA expression was accompanied by reduced nitrate/nitrite levels in some tissues (i.e., kidney and pancreas). Therefore, whereas nitrate deficiency in T2D was associated with increased sialin mRNA expression in some tissues (i.e., adrenal gland, eAT, stomach, soleus muscle, and liver) due to a compensatory response, it was associated with decreased sialin mRNA expression in some other tissues. Although not completely understood, high apical expression of sialin in the kidney distal tubule cells of female mice^74^, male pigs, and male humans^31^, suggests that sialin may contribute to renal reabsorption of nitrate^75^. Therefore, it is expected that decreased sialin mRNA expression in the kidney would be accompanied by reduced nitrate and nitrite levels. However, our data cannot explain the causality between nitrate/nitrite deficiency and sialin expression, and this issue needs further investigation.

As a strength, we used an HFD/low-dose STZ model of T2D, which mimics the pathophysiology of T2D in humans by inducing long-lasting and stable hyperglycemia, insulin resistance, relative hyperinsulinemia, hypertriglyceridemia and also it is sensitive to glucose-lowering effects of metformin and troglitazone^41,76^. In this model, consumption of HFD induces insulin resistance, and subsequently, administration of STZ at a low dose causes partial destruction of pancreatic β-cells^41^ by apoptosis^77^. Increased apoptosis is responsible for impaired insulin secretion in pancreatic β-cell in both humans and rats^77–79^. We used low dose of STZ along with HFD, as it has been reported that the animals fed only with HFD for 2^76^, 4^80^, 16^81^, and 36^82^ weeks progressed insulin resistance but not hyperglycemia.

As a limitation, we used ketamine/xylazine to anesthetize rats that can increase blood glucose in normal (~ 40%)^83^ and diabetic (~ 60%)^84^ rats. However, the hyperglycemic effect of ketamine/xylazine is acute (observed only 6–22 min after IP injection^83^) and mainly observed in fed, not fasted rats^84–86^. We measured our serum glucose values after overnight fasting and therefore it is unlikely that our data was affected by use of ketamine/xylazine.

In conclusion, our results indicated altered sialin mRNA expression, as a nitrate transporter, in the main tissues of male T2D rats. It seems that increased sialin mRNA expression in some tissues (i.e., soleus muscle, adrenal gland, liver, and eAT) may act as a compensatory mechanism to counteract reduced NO bioavailability; however, this hypothesis does not explain decreased mRNA expression of sialin in the pancreas and kidney. Since sialin can play an important role in the physiological regulation of systemic nitrate–nitrite–NO balance^31^, these data may have implications for future NO-based treatment of T2D, which has been suggested to be a cost-effective approach^87^. Further studies are needed to elucidate the role of sialin in the pathophysiology of T2D.

## Supplementary Information

Supplementary Information.

## Data Availability

The datasets used and/or analysed during the current study available from the corresponding author on reasonable request.

## References

[1] IDF, International Diabetes Federation (2019). IDF Diabetes Atlas.

[2] Carlström M (2010). Dietary inorganic nitrate reverses features of metabolic syndrome in endothelial nitric oxide synthase-deficient mice. Proc. Natl. Acad. Sci..

[3] Lundberg JO, Carlström M, Larsen FJ, Weitzberg E (2011). Roles of dietary inorganic nitrate in cardiovascular health and disease. Cardiovasc. Res..

[4] Ghasemi A (2022). Quantitative aspects of nitric oxide production from nitrate and nitrite. EXCLI J..

[5] Lundberg JO, Gladwin MT, Weitzberg E (2015). Strategies to increase nitric oxide signalling in cardiovascular disease. Nat. Rev. Drug Discov..

[6] Kapil V (2020). The noncanonical pathway for in vivo nitric oxide generation: The nitrate–nitrite–nitric oxide pathway. Pharmacol. Rev..

[7] Gilliard CN (2018). Effect of dietary nitrate levels on nitrate fluxes in rat skeletal muscle and liver. Nitric Oxide Biol. Chem..

[8] Schiffer TA, Lundberg JO, Weitzberg E, Carlström M (2020). Modulation of mitochondria and NADPH oxidase function by the nitrate-nitrite-NO pathway in metabolic disease with focus on type 2 diabetes. Biochim. Biophys. Acta BBA Mol. Basis Dis..

[9] Kina-Tanada M (2017). Long-term dietary nitrite and nitrate deficiency causes the metabolic syndrome, endothelial dysfunction and cardiovascular death in mice. Diabetologia.

[10] Muhammed SJ, Lundquist I, Salehi A (2012). Pancreatic β-cell dysfunction, expression of iNOS and the effect of phosphodiesterase inhibitors in human pancreatic islets of type 2 diabetes. Diabetes Obes. Metab..

[11] Lin KY (2002). Impaired nitric oxide synthase pathway in diabetes mellitus: Role of asymmetric dimethylarginine and dimethylarginine dimethylaminohydrolase. Circulation.

[12] Bahadoran Z, Ghasemi A, Mirmiran P, Azizi F, Hadaegh F (2015). Beneficial effects of inorganic nitrate/nitrite in type 2 diabetes and its complications. Nutr. Metab..

[13] Lundberg JO, Weitzberg E (2009). NO generation from inorganic nitrate and nitrite: Role in physiology, nutrition and therapeutics. Arch. Pharmacal Res..

[14] Gheibi S, Jeddi S, Carlström M, Gholami H, Ghasemi A (2018). Effects of long-term nitrate supplementation on carbohydrate metabolism, lipid profiles, oxidative stress, and inflammation in male obese type 2 diabetic rats. Nitric Oxide Biol. Chem..

[15] Varzandi T (2018). Effect of long-term nitrite administration on browning of white adipose tissue in type 2 diabetic rats: A stereological study. Life Sci..

[16] Nyström T (2012). Inorganic nitrite stimulates pancreatic islet blood flow and insulin secretion. Free Radic. Biol. Med..

[17] Ghasemi A, Afzali H, Jeddi S (2022). Effect of oral nitrite administration on gene expression of SNARE proteins involved in insulin secretion from pancreatic islets of male type 2 diabetic rats. Biomed. J...

[18] Ohtake K (2015). Dietary nitrite supplementation improves insulin resistance in type 2 diabetic KKAy mice. Nitric Oxide Biol. Chem..

[19] Jiang H (2014). Dietary nitrite improves insulin signaling through GLUT4 translocation. Free Radic. Biol. Med..

[20] Khoo NKH (2014). Nitrite augments glucose uptake in adipocytes through the protein kinase A-dependent stimulation of mitochondrial fusion. Free Radic. Biol. Med..

[21] Roberts LD (2015). Inorganic nitrate promotes the browning of white adipose tissue through the nitrate–nitrite–nitric oxide pathway. Diabetes.

[22] Ghasemi A, Gheibi S, Kashfi K, Jeddi S (2023). Anti-oxidant effect of nitrite in the pancreatic islets of type 2 diabetic male rats. Iran. J. Basic Med. Sci..

[23] Ip YK (2020). The fluted giant clam (*Tridacna squamosa*) increases nitrate absorption and upregulates the expression of a homolog of SIALIN (H+: 2NO_3_^−^ cotransporter) in the ctenidium during light exposure. Coral Reefs.

[24] Verheijen FW (1999). A new gene, encoding an anion transporter, is mutated in sialic acid storage diseases. Nat. Genet..

[25] Aula N (2000). The spectrum of SLC17A5-gene mutations resulting in free sialic acid-storage diseases indicates some genotype-phenotype correlation. Am. J. Hum. Genet..

[26] Zárybnický T, Heikkinen A (2021). Modeling rare human disorders in mice: The Finnish disease heritage. Cells.

[27] He M (2011). Postnatal expression of sialin in the mouse submandibular gland. Arch. Oral Biol..

[28] Reimer RJ (2013). SLC17: A functionally diverse family of organic anion transporters. Mol. Asp. Med..

[29] Reimer RJ, Edwards RH (2004). Organic anion transport is the primary function of the SLC17/type I phosphate transporter family. Pflug. Arch..

[30] Courville P, Quick M, Reimer RJ (2010). Structure-function studies of the SLC17 transporter sialin identify crucial residues and substrate-induced conformational changes. J. Biol. Chem..

[31] Qin L (2012). Sialin (SLC17A5) functions as a nitrate transporter in the plasma membrane. Proc. Natl. Acad. Sci. USA.

[32] Qin L, Wang S (2022). Protective roles of inorganic nitrate in health and diseases. Curr. Med..

[33] Piknova B, Park JW, Kwan Jeff Lam K, Schechter AN (2016). Nitrate as a source of nitrite and nitric oxide during exercise hyperemia in rat skeletal muscle. Nitric Oxide Biol. Chem..

[34] Feng X (2021). Dietary nitrate supplementation prevents radiotherapy-induced xerostomia. eLife..

[35] Park JW (2020). Potential roles of nitrate and nitrite in nitric oxide metabolism in the eye. Sci. Rep..

[36] Srihirun S (2020). Nitrate uptake and metabolism in human skeletal muscle cell cultures. Nitric Oxide Biol. Chem..

[37] Yin J (2006). Hypoxic culture induces expression of sialin, a sialic acid transporter, and cancer-associated gangliosides containing non-human sialic acid on human cancer cells. Can. Res..

[38] El-Ashmawy NE, Khedr NF, Sallam M, Nossier AI (2022). Effect of activation of liver X receptor alpha on cardiac & hepatic ABCC10 and SLC17A5 drug transporters in hypercholesterolemic rat model. Biochem. Biophys. Res. Commun..

[39] Ahmadi-Noorbakhsh S (2021). Guideline for the care and use of laboratory animals in Iran. Lab Anim..

[40] Percie du Sert N, Hurst V, Ahluwalia A (2020). The ARRIVE guidelines 2.0: Updated guidelines for reporting animal research. PLoS Biol..

[41] Gheibi S, Kashfi K, Ghasemi A (2017). A practical guide for induction of type-2 diabetes in rat: Incorporating a high-fat diet and streptozotocin. Biomed. Pharmacother..

[42] Miranda KM, Espey MG, Wink DA (2001). A rapid, simple spectrophotometric method for simultaneous detection of nitrate and nitrite. Nitric Oxide Biol. Chem..

[43] Ghasemi A, Hedayati M, Biabani H (2007). Protein precipitation methods evaluated for determination of serum nitric oxide end products by the Griess assay. JMSR.

[44] Navarro-Gonzalvez JA, García-Benayas C, Arenas J (1998). Semiautomated measurement of nitrate in biological fluids. Clin. Chem..

[45] Faraldi M (2022). A novel methodological approach to simultaneously extract high-quality total RNA and proteins from cortical and trabecular bone. Open Biol..

[46] Foss DL, Baarsch MJ, Murtaugh MP (1998). Regulation of hypoxanthine phosphoribosyltransferase, glyceraldehyde-3-phosphate dehydrogenase and beta-actin mRNA expression in porcine immune cells and tissues. Anim. Biotechnol..

[47] Gong H (2016). Evaluation of candidate reference genes for RT-qPCR studies in three metabolism related tissues of mice after caloric restriction. Sci. Rep..

[48] Gholami K, Loh SY, Salleh N, Lam SK, Hoe SZ (2017). Selection of suitable endogenous reference genes for qPCR in kidney and hypothalamus of rats under testosterone influence. PLoS ONE..

[49] Dai Y (2021). Identification and validation of reference genes for RT-qPCR analysis in fetal rat pancreas. Reprod. Toxicol..

[50] Brattelid T (2010). Reference gene alternatives to Gapdh in rodent and human heart failure gene expression studies. BMC Mol. Biol..

[51] Julian GS, de Oliveira RW, Perry JC, Tufik S, Chagas JR (2014). Validation of housekeeping genes in the brains of rats submitted to chronic intermittent hypoxia, a sleep apnea model. PLoS ONE.

[52] Lu X (2021). Determination of the panel of reference genes for quantitative real-time PCR in fetal and adult rat intestines. Reprod. Toxicol..

[53] Perez LJ (2017). Validation of optimal reference genes for quantitative real time PCR in muscle and adipose tissue for obesity and diabetes research. Sci. Rep..

[54] Marcisz, M. Practical application of coefficient of variation. *Proceedings of the 13th International Congress on Energy and Mineral Resources (CIERM 2013)* 202–208 (2013).

[55] Pfaffl MW, Horgan GW, Dempfle L (2002). Relative expression software tool (REST) for group-wise comparison and statistical analysis of relative expression results in real-time PCR. Nucleic Acids Res..

[56] Rao X, Huang X, Zhou Z, Lin X (2013). An improvement of the 2ˆ(-delta delta CT) method for quantitative real-time polymerase chain reaction data analysis. Biostat. Bioinform. Biomath..

[57] Shokri M (2021). Effect of nitrate on gene and protein expression of nitric oxide synthase enzymes in insulin-sensitive tissues of Type 2 diabetic male rats. Endocr. Metab. Immune Disord. Drug Targets.

[58] Bulhak AA (2009). PPAR-α activation protects the type 2 diabetic myocardium against ischemia-reperfusion injury: Involvement of the PI3-Kinase/Akt and NO pathway. Am. J. Physiol. Heart Circ. Physiol..

[59] Bitar MS (2005). Nitric oxide dynamics and endothelial dysfunction in type II model of genetic diabetes. Eur. J. Pharmacol..

[60] Zhang H (2010). Resveratrol improves left ventricular diastolic relaxation in type 2 diabetes by inhibiting oxidative/nitrative stress: In vivo demonstration with magnetic resonance imaging. Am. J. Physiol. Heart Circ. Physiol..

[61] Siervo M, Stephan BC, Feelisch M, Bluck LJ (2011). Measurement of in vivo nitric oxide synthesis in humans using stable isotopic methods: A systematic review. Free Radic. Biol. Med..

[62] Ghasemi A, Jeddi S (2017). Anti-obesity and anti-diabetic effects of nitrate and nitrite. Nitric Oxide Biol. Chem..

[63] Jansson EA (2008). A mammalian functional nitrate reductase that regulates nitrite and nitric oxide homeostasis. Nat. Chem. Biol..

[64] Venardos K, Zhang WZ, Lang C, Kaye DM (2009). Effect of peroxynitrite on endothelial L-arginine transport and metabolism. Int. J. Biochem. Cell Biol..

[65] Łuczak A, Madej M, Kasprzyk A, Doroszko A (2020). Role of the eNOS uncoupling and the nitric oxide metabolic pathway in the pathogenesis of autoimmune rheumatic diseases. Oxid. Med. Cell. Longev..

[66] Zou MH, Shi C, Cohen RA (2002). Oxidation of the zinc-thiolate complex and uncoupling of endothelial nitric oxide synthase by peroxynitrite. J. Clin. Invest..

[67] McLennan S (1988). Deficiency of ascorbic acid in experimental diabetes: Relationship with collagen and polyol pathway abnormalities. Diabetes.

[68] Wilson R (2017). Inadequate vitamin C status in prediabetes and type 2 diabetes mellitus: Associations with glycaemic control, obesity, and smoking. Nutrients.

[69] Bahadoran Z, Mirmiran P, Carlström M, Ghasemi A (2021). Inorganic nitrate: A potential prebiotic for oral microbiota dysbiosis associated with type 2 diabetes. Nitric Oxide Biol. Chem..

[70] Park JW, Piknova B, Dey S, Noguchi CT, Schechter AN (2019). Compensatory mechanisms in myoglobin deficient mice preserve NO homeostasis. Nitric Oxide Biol. Chem..

[71] Park JW, Thomas SM, Schechter AN, Piknova B (2021). Control of rat muscle nitrate levels after perturbation of steady state dietary nitrate intake. Nitric Oxide Biol. Chem..

[72] Ghasemi A, Jeddi S (2022). Quantitative aspects of nitric oxide production in the heart. Mol. Biol. Rep..

[73] Piknova B, Schechter AN, Park JW, Vanhatalo A, Jones AM (2022). Skeletal muscle nitrate as a regulator of systemic nitric oxide homeostasis. Exerc. Sport Sci. Rev..

[74] Yarovaya N (2005). Sialin, an anion transporter defective in sialic acid storage diseases, shows highly variable expression in adult mouse brain, and is developmentally regulated. Neurobiol. Dis..

[75] Carlström M (2021). Nitric oxide signalling in kidney regulation and cardiometabolic health. Nat. Rev. Nephrol..

[76] Reed MJ (2000). A new rat model of type 2 diabetes: The fat-fed, streptozotocin-treated rat. Metab. Clin. Exp..

[77] Ghasemi A, Khalifi S, Jedi S (2014). Streptozotocin-nicotinamide-induced rat model of type 2 diabetes (review). Acta Physiol. Hung..

[78] Butler AE (2003). Beta-cell deficit and increased beta-cell apoptosis in humans with type 2 diabetes. Diabetes.

[79] Ghasemi A, Jeddi S (2023). Streptozotocin as a tool for induction of rat models of diabetes: A practical guide. EXCLI J..

[80] Tanaka S (2007). High-fat diet impairs the effects of a single bout of endurance exercise on glucose transport and insulin sensitivity in rat skeletal muscle. Metab. Clin. Exp..

[81] Ishii Y (2010). A high-fat diet inhibits the progression of diabetes mellitus in type 2 diabetic rats. Nutr. Res. (New York, NY).

[82] Zhao S (2008). Diet-induced central obesity and insulin resistance in rabbits. J. Anim. Physiol. Anim. Nutr..

[83] Guarino MP, Santos AI, Mota-Carmo M, Costa PF (2013). Effects of anaesthesia on insulin sensitivity and metabolic parameters in Wistar rats. In vivo (Athens, Greece)..

[84] Chen H, Li L, Xia H (2015). Diabetes alters the blood glucose response to ketamine in streptozotocin-diabetic rats. Int. J. Clin. Exp. Med..

[85] Hindlycke M, Jansson L (1992). Glucose tolerance and pancreatic islet blood flow in rats after intraperitoneal administration of different anesthetic drugs. Upsala J. Med. Sci..

[86] Saha JK, Xia J, Grondin JM, Engle SK, Jakubowski JA (2005). Acute hyperglycemia induced by ketamine/xylazine anesthesia in rats: Mechanisms and implications for preclinical models. Exp. Biol. Med..

[87] Lundberg JO, Carlstrom M, Weitzberg E (2018). Metabolic effects of dietary nitrate in health and disease. Cell Metab..

