# Supplementary material for: Altered sialin mRNA gene expression in type 2 diabetic male Wistar rats: implications for nitric oxide deficiency
Source: Sci Rep. 2023 Mar 10;13:4013. doi: 10.1038/s41598-023-31240-4 (PMC10006425; doi:10.1038/s41598-023-31240-4)
Supplement: Supplementary file 1 — Supplementary Information. [file 41598_2023_31240_MOESM1_ESM.docx]

**Supplementary Figure 1:** Changes in tissues’ sialin mRNA expression in type 2 diabetic rats compared to controls.
